# Docosahexaenoic acid in diluent for goat semen cryopreservation

**DOI:** 10.1590/1984-3143-AR2021-0027

**Published:** 2021-10-29

**Authors:** Rosiléia Silva Souza, William Morais Machado, Caline Santana da França, Lopes César Mugabe, Emmanuel Emydio Gomes Pinheiro, Isabella de Matos Brandão Carneiro, Laiara Fernandes Rocha, Larissa Pires Barbosa

**Affiliations:** 1 Programa de Pós-graduação em Ciências Animal, Centro de Ciências Agrárias, Ambientais e Biológicas, Universidade Federal do Recôncavo da Bahia, Cruz das Almas, BA, Brasil

**Keywords:** fatty acids, semen, lipids, goat, cryopreservation

## Abstract

The effects of docosahexaenoic acid (DHA) in the diluent for cryopreservation of goat semen on seminal quality and the optimal levels to be used were evaluated. After collection, semen was pooled and physically evaluated, then divided into four aliquots with different DHA levels in the diluent: 0, 10, 20, and 30 ng mL^-1^. The semen was cryopreserved in a TK 3000® freezing machine and then thawed for assessment at 37 °C. Sperm motility and vigor, membrane integrity, acrosomal integrity, mitochondrial activity, and sperm chromatin compaction were evaluated after thawing. A completely randomized design was used. For normally distributed variables, ANOVA and regression analysis were used to test for differences between treatments, and for non-parametric data, the Kruskal Wallis test was used at the 5% significance level. There were no differences among groups in terms of membrane integrity, acrosomal integrity, or chromatin compaction. There was a decrease in class I mitochondrial activity with increasing DHA level (P<0.05), but no differences in classes II, III, and IV (P>0.05). The inclusion of 10 to 30 ng mL^-1^ of DHA in the diluent did not result in improvements in seminal quality parameters after thawing, with some impairment observed in the mitochondrial activity of the sperm cells.

## Introduction

Seminal cryopreservation has contributed to the expansion of reproductive biotechniques, such as artificial insemination and *in vitro* fertilization. However, the process promotes changes in the composition of PUFAS in the sperm plasma membrane, decreasing motility, viability, and acrosomal integrity (Martinez-Soto et al., 2013).

According to Nasiri et al. (2012), DHA added to the diluent is incorporated into the plasma membrane of sperm cells. Safarinejad et al. (2010) affirmed that the presence of DHA in the sperm plasma membrane increases cryogenic tolerance and maintains the physiological properties of the lipid bilayer.

Thus, the objective of this study was to evaluate the effects of DHA inclusion in the diluent for cryopreservation of goat semen and determine the optimal level of use.

### Material and methods

Five adult male Anglo-Nubian goats were used in the experiment, with an average age of 3.30±1.67 years, body condition score of 3.25±0.50, and body weight of 63.06±18.24 kg. Over a three-week period, the animals were subjected to an intensive production system, and were supplied with Tifton-85 hay (*Cynodon* sp.), a concentrated feed mixture based on corn bran and soy [formulated according to the NRC (2007)], and *ad libitum* water. The animals were fed twice a day, with daily leftovers of between 10% and 20%.

Sample collections were performed twice a week using the artificial vagina technique, using a female in estrus as a mannequin, totaling five ejaculates per animal. Physical characteristics of the ejaculates were evaluated *in vitro* using differential phase interference microscopy (Olympus®, Tóquio, Japan). Samples with sperm motility ≥70% and vigor ≥3 [Colégio Brasileiro de Reprodução Animal (CBRA, 2013)] were grouped to form a pool.

After formation of the pool, physical and morphological characteristics were reassessed. Samples that presented values within the standards required by CBRA (2013) were diluted and divided into four treatment groups (G) with different DHA levels: G1 (n=5): 0 ng mL^-1^; G2 (n=5): 10 ng mL^-1^; G3 (n=5): 20 ng mL^-1^; and G4 (n=5): 30 ng mL^-1^. Samples were mixed with 0.2 mmol of alpha-tocopherol, diluted in 0.05% ethanol solution in an egg yolk-citrate diluent.

Sperm concentration was assessed after dilution of 20 μL of semen in 8 mL of 10% formaldehyde-saline. Sperm counting was performed in a Neubauer chamber CBRA (2013).

To evaluate sperm morphology, two hundred cells were counted and evaluated under immersion using differential phase interference microscopy (CBRA, 2013).

DHA treatment stock solutions (DHA, Sigma-Aldrich®, Brazil) were prepared considering its molecular weight of 328.49 g/mol and were diluted in 100 mL of 0.05% ethanol solution. For each group, 0.2 mmol of vitamin E (alpha-tocopherol, Sigma-Aldrich ®, Brazil) was diluted in 0.05% ethanol solution.

A final seminal dilution was conducted to obtain a concentration of 100 million spermatozoa per dose, using 0.25 mL straws (Minitüb®, Tiefenbach, Germany). The straws were sealed and sent for cryopreservation in a TK 3000® freezing machine (TK Freezing Technology, Brazil), using the curve for goats (P4S2).

The functional integrity of the plasma membrane was evaluated using the hyposmotic test (HOST). Two hundred sperm cells were classified under immersion using phase contrast microscopy, for the presence (or absence) of a bent tail, according to Kumi-Diaka (1993). The number of sperm reactive to HOST was calculated using the formula described by Melo and Henry (1999).

Acrosomal membrane integrity was assessed using the Cerovsky (1976) method with Congo Red coloring. Seminal smears were prepared using an aliquot from each thawed straw.

Sperm mitochondrial activity was evaluated by incubating 20 μL of each thawed sample with 20 μL of 3.3′-diaminobenzidine (DAB) (Sigma-Aldrich®, Brazil) and 1.0 mg mL^-1^ of phosphate buffered saline (PBS) at 37 °C for 60 min in the absence of light. Using a differential phase interference microscope (Olympus®), 200 spermatozoa per slide were assessed and classified according to Hrudka (1987).

The Beletti and Mello (2004) protocol was used to assess sperm chromatin compaction. Smears were made with an aliquot of each thawed straw. Five hundred spermatozoa were evaluated per slide using light microscopy under immersion and were classified as intact or fragmented chromatin.

A completely randomized design was used. Data were evaluated for normality using the Shapiro-Wilk test. Variables that presented a normal distribution were analyzed using ANOVA and regression, and non-parametric data were assessed using the Kruskal Wallis test at the 5% significance level. SPSS version 23 (IBM SPSS Statistics for Windows, Version 23.0. Armonk, NY) was used for all analyses.

The experiment was performed at the Federal University of Recôncavo of Bahia (UFRB), Cruz das Almas, Bahia, Brazil (12º40'12″ S, 39º06'07″ W), during the winter. The area has average annual rainfall of 1224 mm, average relative air humidity of 80%, and an average annual temperature of 24.5 ºC (IBGE, 2000). The study was approved by the Ethics Committee of the UFRB (23007.006635/ 2014–60).

## Results

There was no difference between treatments in progressive sperm motility (64.50±6.66%) or sperm vigor (2.05±0.57) after thawing (P>0.05). The average found for sperm progressive motility in fresh semen in the study was 86.00±4.18 and sperm vigor was 3.9±0.41.

In this study, estimated losses related to the cryopreservation process were 21.5% and 1.9% of progressive motility and vigor, respectively. The selected semen (pool) for the cryopreservation process presented average progressive motility of greater than 70% (86.00±4.18) and average sperm vigor greater than 3.0 (3.9±0.41).

There were no differences in the membrane integrity of cryopreserved semen between treatments, with averages of 37.47±9.75% for reactive spermatozoa and 62.52±9.75% for non-reactive spermatozoa (P>0.05).

There was no difference in the acrosomal integrity (P>0.05) of cryopreserved semen between treatments. The results showed average acrosomal integrity of 73.35±13.92. The results for irregular acrosome were 10.12±6.19. For the partial acrosome detachment were12.38±7.46 and for the total acrosome detachment were 4.15±2.88.

There were no differences in mitochondrial activity between treatments in classes II, III, and IV (P>0.05), with averages of 20.3±3.93%, 16.72±5.66%, and 39.4±10.46%, respectively. There was a significant decrease in mitochondrial activity in class I with DHA level in the diluent. Groups G1–4 showed average mitochondrial activity of 29.10±4.70, 22.70±2.77, 20.10±2.81, and 21.80±4.89%, respectively (P<0.05).

There was no difference in chromatin compaction (P>0.05) of goat semen between treatments, with averages of 99.06±0.90% for intact chromatin and 0.94±0.90% for fragmented chromatin.

## Discussion

These values are superior to the 30% for progressive sperm motility and 2.0 for sperm vigor recommended by the CBRA (2013) for cryopreserved goat semen.

Machado et al. (2018) incorporated up to 4% fish oil and a fixed dose of ascorbic acid in the diluent for cryopreservation of goat semen. They found an increase in progressive sperm motility with increasing levels of fish oil, indicating that the addition of fish oil (DHA source) was beneficial to the cryopreservation process and improved seminal quality after thawing.

There were no differences in the membrane integrity of cryopreserved goat semen between treatments. Aguiar et al. (2020) included up to 50 ng mL^-1^ of DHA, with or without Trolox®, in a BotuSemen^®^ diluent for cooling stallion semen. They verified that the structural and functional integrity of the membranes were preserved in a similar way in all dilutors used in the cooling process.

There was no difference in the acrosomal integrity (P>0.05) of cryopreserved goat semen between treatments. Machado et al. (2018) reported improvements in acrosomal integrity with 1% and 4% fish oil as a DHA source in the diluent for cryopreservation of goat semen, indicating that the DHA present in fish oil may have been incorporated into the acrosomal membrane, providing greater resistance to the cryopreservation process.

Mahadevan et al. (1997) reported that mitochondrial activity is related to sperm motility, with the necessary energy for motility and fertilization provided in the form of ATP synthesized through oxidative phosphorylation in the mitochondria.

In this study, the observed negative trend in mitochondrial activity (class I), could be linked to the cryopreservation process and/or the antioxidant concentrations in the diluent, resulting in damage to the plasma membrane and internal sperm structures, including mitochondria.

## Conclusion

We conclude that the inclusion of 10 to 30 ng mL^-1^ of DHA did not improve the seminal quality parameters of post-thaw goat semen, but higher levels impaired sperm cell mitochondrial activity.
